# Increasing recruitment to randomised trials: a review of randomised controlled trials

**DOI:** 10.1186/1471-2288-6-34

**Published:** 2006-07-19

**Authors:** Judith M Watson, David J Torgerson

**Affiliations:** 1 York Trials Unit, Department of Health Sciences, University of York, York, YO10 5DD, UK

## Abstract

**Background:**

Poor recruitment to randomised controlled trials (RCTs) is a widespread and important problem. With poor recruitment being such an important issue with respect to the conduct of randomised trials, a systematic review of controlled trials on recruitment methods was undertaken in order to identify strategies that are effective.

**Methods:**

We searched the register of trials in Cochrane library from 1996 to end of 2004. We also searched Web of Science for 2004. Additional trials were identified from personal knowledge. Included studies had to use random allocation and participants had to be allocated to different methods of recruitment to a 'real' randomised trial. Trials that randomised participants to 'mock' trials and trials of recruitment to non-randomised studies (e.g., case control studies) were excluded. Information on the study design, intervention and control, and number of patients recruited was extracted by the 2 authors.

**Results:**

We identified 14 papers describing 20 different interventions. Effective interventions included: telephone reminders; questionnaire inclusion; monetary incentives; using an 'open' rather than placebo design; and making trial materials culturally sensitive.

**Conclusion:**

Few trials have been undertaken to test interventions to improve trial recruitment. There is an urgent need for more RCTs of recruitment strategies.

## Background

Recruitment to randomised trials can be very poor [1,2]. A recent survey of corresponding authors of randomised trials published between the years 2000 and 2001 in the Lancet or BMJ found that nearly 60% had either failed to meet their recruitment target or required an extended recruitment period [3].

Poor or slow recruitment will have some or all of the following consequences. First, the possibility of incurring a Type II error increases if the sample size target is not met (i.e. erroneously concluding there is no significant difference between treatment groups). Second, even if the required sample size is met, if recruitment is slow, the trial may need to be extended which increases its costs. Third, late recruitment maintains the level of uncertainty about treatment effectiveness and delays the time a potentially effective therapy can be offered to the general population or increases the time that people are exposed to an ineffective or dangerous treatment. Finally, slow acquisition of trial evidence can reduce the investment in the conduct of trials by funding agencies, who may consequently prefer to invest in less reliable but more rapid approaches to evaluation.

There have been two recent systematic reviews looking at methods to increase recruitment to clinical trials. A Cochrane review [4] identified 15 controlled trials of interventions to increase recruitment and a review looking at the factors limiting the quality, number and progress of RCTs sponsored by the HTA [1] found no randomised comparisons of different strategies, only trials that judged the relative effectiveness of a variety of strategies based on their perceived success. However, overall limiting factors were the main focus of the HTA search, not identifying recruitment strategies and this may account for the difference in RCTs identified.

Because poor recruitment is such an important issue with respect to the conduct of randomised trials we decided to undertake a systematic review of controlled trials on recruitment methods in order to add to the findings of the Cochrane review [4] and to establish whether researchers were evaluating recruitment strategies by incorporating them into their research studies, as recommended by the Cochrane review.

## Methods

### Searching

There have been at least two previous reviews on recruitment strategies [1,4]: one of the most comprehensive was sponsored the by the UK NHS HTA programme and did not identify any RCTs of recruitment strategies through 1996 [1]. One of us (JW) adapted a search strategy previously used by the authors of the NHS HTA report on trial recruitment. This was used to search the register of trials in Cochrane library between the years 1996 to end of 2004. The Cochrane review search strategy was re-ran for 2002–2004. We also searched Web of Science, Cochrane Central Register of Controlled Trials, Medline, CINAHL and Embase using the key words "recruitment strategy" and "recruitment strategies" for 2004. Additional trials were identified from personal knowledge. No attempt was made to contact authors for unpublished data, nor was any extensive hand searching conducted.

### Selection

The abstracts of the search results were read by one of the authors, and potentially relevant papers identified. Any uncertainties were discussed by both authors and full text versions obtained if necessary to resolve differences. Our inclusion criteria were as follows. The study had to use random allocation and participants had to be allocated to different methods of recruitment to a 'real' randomised trial. We excluded trials that randomised participants to 'mock' trials and trials of recruitment to non-randomised studies (e.g., case control studies). Full text versions were then obtained and both authors read the potentially relevant papers.

### Validity assessment

Four of the papers gave no information as to how they carried out the randomisation procedure, whilst the other ten used computer programmes in some way. Allocation concealment was thought to be adequate in seven of the included papers, but there was insufficient information given in the remaining papers for a clear decision to be made.

### Data extraction

We extracted information on the study design, details of the recruitment strategies being compared, and number of patients recruited. These data were extracted by the two authors and any disagreements were resolved through discussion.

### Data analysis

Two trials evaluated the use of placebo versus an open design. Because they were methodologically similar we pooled these in a meta-analysis using Revman.

## Results

**Figure 1:**
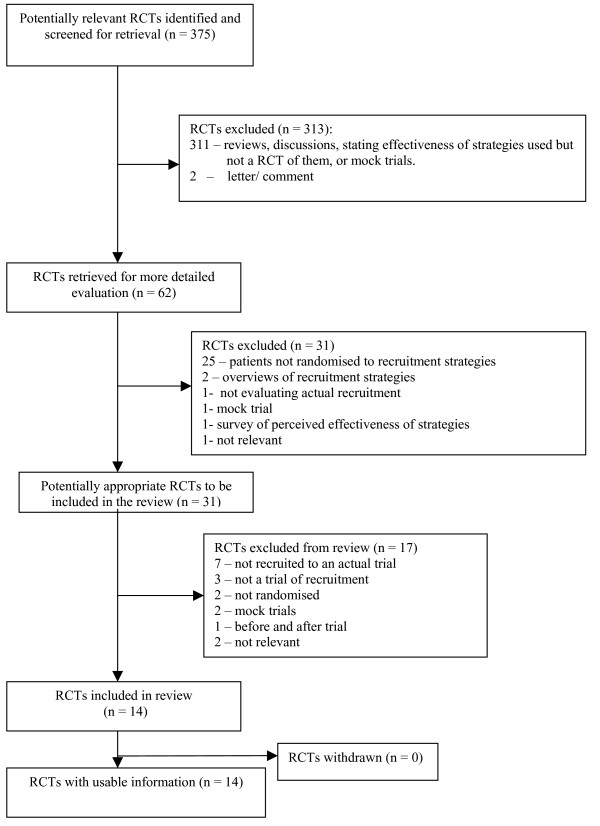
Flow diagram of the retrieval of papers included in this review.

**Figure 2:**
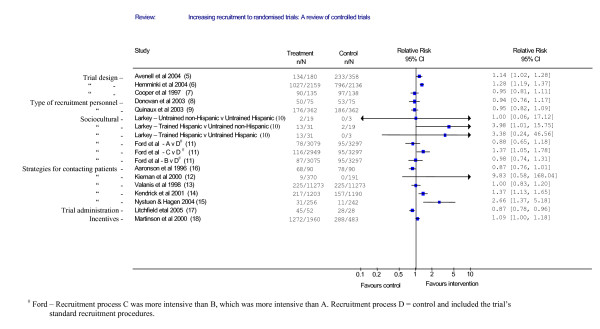
Comparison: Effectiveness of strategies to increase recruitment.

We identified 62 possibly relevant studies on the basis of title and abstracts and 14 studies were included. The 14 papers described trials of 20 methods to increase recruitment rates. Figure 1 shows the flow of the retrieval of papers and reasons for exclusion. Additional file: 1 describes the characteristics of the included trials and the interventions that were evaluated, whilst Table 1 summarises the types of interventions the studies looked at. In Figure 2 we present a forest plot. As the figure shows a number of the studies showed statistically significant benefits of various interventions on recruitment.

**Table 1:** Summary of the recruitment strategies looked at by the included studies.

**Area**	**Strategy**	Reference
*Trial Design*	Open versus blind	5, 6
	Patient preference design	7
*Type of recruitment personnel*	Nurses versus doctors	8
	Trial Co-ordinator	10
*Socio-cultural*	Lay advocates	11
	Address barriers to minority recruitment	12
*Strategies for contacting patients*	Quantity of information given	9
	Mailing strategies	13
	Advance postcard	14
	With or without questionnaire	15
	Telephone reminder	16
*Trial administration*	Internet system versus paper	17
*Incentives*	Monetary incentive	18

### Trial design

**Figure 3:**
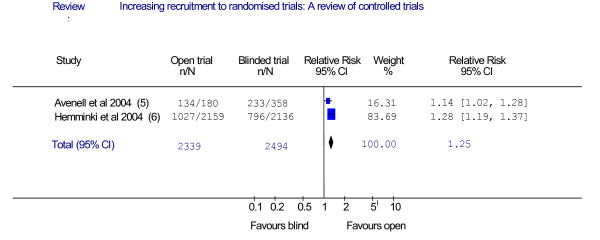
Comparison: Open arm of trial compared with blind arm of trial.

Two trials looked at the use of an open versus a placebo control to increase recruitment (Figure 3) [5,6]. The trials showed that the use of blinding is associated with poorer recruitment [5,6] and one showed higher retention rates using the 'open' design (where both patient and clinician know the given treatment) [5]. Figure 3 shows the statistically significant increase in recruitment when an open design was used. The pooled odds ratio (OR) of 1.53 (95% CI 1.36 to 1.72) is highly statistically significant (p < 0.00001).

One trial looking at the use of a patient preference design found no difference in numbers recruited to the randomised arms of the trial [7]. Although the authors found that more patients initially participated in the partially randomised patient preference design, there was no difference in the numbers who actually agreed to be randomised were not statistically significant.

### Type of recruitment personnel

One UK trial looked at the use of different health care professionals to increase recruitment rates, where nurses were compared with consultants in the context of a prostate cancer trial [8]. A small, but not statistically significant difference was found in favour of consultant recruitment.

We also identified a single trial looking at the use of a trial co-ordinator to visit clinical sites to recruit into a French oncology trial [9]. This did not appear to have a statistically significant effect.

### Sociocultural barriers

A trial based in the USA looked at use of lay advocates to help with the recruitment of Hispanic women into a Women's Health Initiative study [10]. These advocates were women who were already enrolled in the study and were then randomised to be trained or not. The women were asked to distribute brochures to women within their area and promote recruitment to the study. In addition, a further control group of untrained non-Hispanic white (Anglo) women was formed. The authors found that trained Hispanic women (or *Embajadoras*) were more successful at referring and enrolling women into the trial than untrained Hispanic (but not statistically significantly) or Anglo women. Similarly, another trial in the USA looked at a range of interventions among African Americans to increased recruitment rates for cancer screening trials [11]. In this large 4-armed study, 3 enhanced recruitment approaches were used including using recruiters from the same ethnic background. Only one approach, recruitment via a local church, showed a statistically significant increase in recruitment, although this increase was only 1% in absolute terms.

### Strategies for contacting patients

Mailing strategies were also examined, with one trial comparing the recruitment yield of three direct mailing strategies [12], where they found that personalized letters improved response rates more than a flyer alone, although not significantly, and a cultural specific personalised letter was no more effective than a generic one. Another looked at alerting recipients by sending an advance postcard one week prior to mailing a full recruitment pack [13], but was found to have no statistically significant effect on the percentage randomised. A third trial compared response and recruitment rates to their injury prevention trial according to whether the invitation to participate was sent out with or without a home safety questionnaire [14], and it was found that both rates significantly increased with the inclusion of the questionnaire. One trial randomised non-responding potential trial participants to receive telephone reminders two weeks after receiving their initial letter of invitation, whilst the controls received no reminder, and it was found that the telephoning significantly increased the number of additional subjects recruited to the trial [15]. Furthermore, an oncology trial in the USA, looked at the use of oncology nurses to provide additional trial information, to that provided by the oncologists. The intervention group showed a non-statistically significant decline in recruitment rates [16].

### Trial administration

One trial compared the use of an internet system to record and collect trial patient data against commonplace paper collection [17] by randomising centres to either method. Whilst benefits did appear to be seen in the efficiency of data collection in site using the internet system, the difference in the numbers of patients recruited per centre between the two group was statistically significant, but in favour of the paper method.

### Incentives

An intervention trial to reduce smoking involving the recruitment of adolescents [18] found that monetary incentives significantly improved response rates and had a positive effect on their willingness to participate further and the proportion agreeing to be contacted about a planned intervention was significantly higher.

## Discussion

Despite the paucity of evidence, this review has highlighted a few approaches that could be considered when designing and implementing a study.

It was found that sociocultural specific interventions have the potential to increase recruitment when recruiting a particular racial or ethnic group. Also, simple strategies such as personalised letters, inclusions of questionnaires, telephone reminders and monetary incentives were seen be effective. Trials using a patient preference design found no improvement in recruitment, nor did those implementing monitoring visits to trial sites, using nurses instead of doctors to recruit or those using an internet data capture system as opposed to a paper based one. Placebo use was detrimental to recruitment rate.

### Strategies proven to improve recruitment

Sociocultural factors are often cited as being important considerations in trial design and one trial here sought to overcome such barriers to trial participation among African Americans [11]. African-American men were randomised to a control group or one of three increasingly intensive intervention arms that used different combinations of mail, telephone and African-American church-based recruitment. The racial, ethnic and language backgrounds of the research team were all similar to those of the potential study participants. Each of the interventions designed to address the main areas previously identified as barriers to the recruitment of minority groups to clinical trials. However, although the most intensive intervention was the most successful, it only managed a 1% greater recruitment yield than the control arm, and raises question as to whether such intensive approaches are worth the resultant extra numbers recruited.

Alternatively, the success of the trained Hispanic women [10] suggest the use of culturally related recruitment methods may be effective at encouraging enrolment. However, although the authors do acknowledge that their study is weakened by the absence of a trained non-Hispanic group, this approach does appear worth considering for trialists.

Despite these cautions, there does appear to be an advantage in using cultural-specific strategies to improve recruitment to trials involving specific ethnic groups.

Trials with 'open' designs also appear to benefit recruitment. The practice of 'blinding' is used by many drug trials as it guards against a number of biases, such as ascertainment bias. However, their use is not pragmatic in the sense that in the 'real' world doctors and patients know what treatment they give and receive. There may be a role, certainly in confirmatory trials, to design these as open randomised trials in order to increase their generalisability, but the number of trials where such a design is possible may be limiting.

The simpler recruitment approaches such as telephone reminders, where almost three times the number were recruited compared to the no-reminder group, and inclusion of a questionnaire also showed an increase in numbers recruited (18% of those invited compared to 13.2% invited without the questionnaire). Therefore, trialists might consider ensuring such straightforward methods are adequately budgeted in for in their trial grant applications.

Martinson et al found that monetary incentives improved response rates amongst adolescents, had a positive effect on their willingness to be contacted about future intervention, thereby increasing the potential numbers who could be recruited. As this strategy has been identified before as an effective way of improving response rates to postal questionnaires [19], if it can also improve recruitment it is a worthwhile for trialists' consideration.

The apparent effectiveness of the above strategies, however, is based only on single studies of each method (except the 'open' design, with only two trials) and their inclusion in future trials is very much dependent on individual trial design. Therefore, if suitable, it may prove worthwhile for researchers to consider the inclusion of one of the above strategies. In turn, if included as a randomised element of the study, such trials would be adding to the evidence on the effectiveness of the strategy.

### Strategies that may potentially improve recruitment

There were a number of strategies identified that showed potential, but would benefit from further research to decide one way or another.

Looking at the person undertaking the recruitment, the difference between a nurse and a doctor was not statistically significant. However, in addition to establishing that nurses are no less effective as surgeons in recruiting patients, Donovan and colleagues found that the nurses were the more cost effective option. As cost is always an important factor, the use of staff other than the clinician could be considered in some trials.

Interestingly, although it is quite common for research staff to visit the trial sites in order to encourage recruitment, in the case of trial co-ordinators doing so, the one trial identified here found no evidence of any benefit [9]. Given the large time investment in making such centre visits, it would be worthwhile repeating this single study to either confirm or refute its findings.

It was seen that personalised letters did improve response rates and thereby the numbers that could be potentially recruited, but the effect was not statistically significant [12]. The authors acknowledge the need for the study to be replicated with a larger sample, but the method does sound promising and has the added benefit of being a relatively simple strategy to implement.

With trial administration procedures playing a large part during trial, a pre-trial internet system studied by Litchfield et al did not show any difference in the number of patients recruited compared to the usual paper method. However there were gains seen in efficiency with its use. These were seen in the time from last patient completing the study to the database being released, data being entered more quickly and queries being resolved earlier.

The above strategies demonstrated the potential to have an effect on recruitment but not one that was large enough to warrant a recommendation for their use. However, as with those that were shown to be effective, the evidence of the effectiveness of these strategies is very limited, with additional and larger trials incorporating them into their design necessary to allow definite recommendations to be made.

### Strategies that do not appear to improve recruitment

A number of strategies did appear to be far less successful, such as sending an advance postcard before mailing a full recruitment pack [13], where although a higher rate of questionnaire return and completion was seen, it did not increase significantly increase the percentage randomised.

Within patient preference trials, patients may be placed within one of three groups according to their preferences: 1) patients with no strong preference and consent to randomisation; 2) patients with a preference but still consent to randomisation; and 3) patients who refuse randomisation and choose their preferred treatment [20]. This is in an attempt to prevent the bias that can occur when patients do not receive their preferred treatment option and could make clinical trials more attractive to those patients who are apprehensive of randomisation. Alternatively, a partially randomised design, as done by Cooper et al, where patients' preferences are identified before randomisation, with all consenting patients randomised [21]. As Torgerson and Sibbald state, patient preference trials are not meant to replace randomised trials, but to complement them, although measuring a patient's preference maintains all the advantage of a RCT, but has the benefit of allowing for interactions between preference and outcome to be assessed [20]. However, Cooper et al discovered no increase in the number of patients randomised [7].

Aaronson and colleagues found their telephone based nursing intervention did not increase the numbers recruited. In fact the intervention group showed a decline in recruitment rates. However, it was effective in increasing some aspects of patients' awareness and understanding of important issues regarding participation in clinical trials, which may benefit participation in the long term.

These apparently non-effective strategies, however, are based on the evidence of single trials. It would therefore be more reassuring to have additional trials to back up this scant evidence before making definitive recommendations to not implement any of these strategies.

Poor recruitment to clinical trials is a major threat to their validity. Low recruitment leads to loss of statistical power and reduces the generalisability of the study to the wider clinical population. Many trialists use unproven methods to try and improve recruitment to studies. Ironically many trialists rely on before and after methods to assess the effectiveness of interventions. For example, Donovan and colleagues claim to have increased recruitment rates to a prostate cancer trial by the incorporation of qualitative methods into the recruitment process [22]. However, this intervention was not subjected to a controlled trial and therefore could have been confounded by regression to the mean, temporal changes or simple selection effects. Therefore, it is crucial that recruitment methods are underpinned by the best possible evidence and this means randomised trials.

One aspect that did not appear in any studies suitable for this review was education or training. In one cluster randomised trial in primary care published after our search strategy [23], a significant increase in recruitment rates to its trial of exercise and manipulation for low back pain, was seen in practices where primary care staff had been educated in the active management of back pain. This was however, an unforeseen effect of the training package and not a randomised evaluation of it as a strategy. Trialists, therefore, in light of the apparent effect might consider offering a professional education package to clinicians in order to boost recruitment.

Interestingly, a method often used to encourage the healthcare professionals to recruit patients (as opposed to encouraging the patients to participate) is payment, which despite being used extensively has limited evidence [24]. In line with the limited number found here, the authors failed to find any controlled studies comparing recruitment rates with and without the financial incentives.

It is acknowledged here that searching for such methodological based papers can be difficult and that we may not have identified all potential papers. We may have been able to broaden the scope of this paper if we had reviewed all papers reporting recruitment strategies and not only those using random allocation, but it is felt that this would have furthered the situation of reported successes of strategies being anecdotal and not based on evidence. It is also unknown how many randomised trials of strategies have not been published due to negative results. Publication would allow trialists to make more informative decisions as to which strategies to use, and would add to the current limited evidence base.

## Conclusion

In conclusion we have identified few trials that have evaluated different recruitment methods demonstrating that little has been done by researchers since the Cochrane review.

Blinding decreases recruitment rates whilst in our own experience, educating clinicians appears to have the effect of increasing recruitment. Cultural specific designs appear to help, as do incentives. There are many papers published which discuss the recruitment strategies they used for recruitment and describe which were the most successful, but there are very few which are actual randomised trials of such strategies. Given this, in order to evaluate them, still more evidence is need before definitive guidance can be given as to which are effective, which can only be gathered if researchers include such evaluations within their trial designs.

## Competing interests

The author(s) declare that they have no competing interests.

## Authors' contributions

DJT suggested the idea for the review. JMW and DJT did the searches and extracted the data from the included papers. JMW, with advice of DJT conducted the analysis. JMW and DJT wrote the paper and both authors have approved the final manuscript.

## Pre-publication history

The pre-publication history for this paper can be accessed here:



## Supplementary Material

Additional file 1 Characteristics of included studies. Click here for file
