# Supplementary material for: Increasing recruitment to randomised trials: a review of randomised controlled trials
Source: BMC Med Res Methodol. 2006 Jul 19;6:34. doi: 10.1186/1471-2288-6-34 (PMC1559709; doi:10.1186/1471-2288-6-34)
Supplement: Additional file 1 — Characteristics of included studies. [file 1471-2288-6-34-S1.doc]

| Additional file 1: Characteristics of included studies | | |  |  | |
| --- | --- | --- | --- | --- | --- |
|  |  |  |  |  | |
| **Study** | **Design** | **Participants** | **Interventions** | **Outcomes** | |
| ***Trial Design*** | | | | | |
| Avenell et al 2004 (5) | Randomised controlled comparison nested within a placebo-controlled trial of nutritional supplementation amongst people aged 70 years or over who had previously sustained a fracture. | People aged 70 years or over who had had an osteoporotic fracture within the last 10 years. | Randomised double-blind placebo-controlled factorial design compared with:  an open trial design which was otherwise identical. Randomisation was 2:1 in favour of the blinded, placebo-controlled design. | Proportions of eligible participants recruited under the two trial designs;  The proportions of participants remaining in the study at one year.  180 randomised (134 consented) to open trial design.  358 randomised (233 consented) to blinded, placebo-controlled trial design | |
| Cooper et al 1997 (7) | Randomised controlled comparison of two clinical trial designs | 309 women referred from a general gynaecological clinic. | Partially randomised patient preference clinical trial design versus:  conventional randomised controlled design. | Overall participation;  Participation in RCT of medical management compared with surgical resection.  273 eligible and 135 randomised to intervention arm (90 eventually participated) and 138 to control (97 participated) | |
| Hemminki et al 2004 (6) | Randomised primary prevention trial of postmenopausal hormone therapy (PHT) | Postmenopausal women. | 4 groups - blind group with PHT; blind group with placebo; non-blind group with PHT; non-blind group with no therapy (control) | Number of women recruited to the groups.  2136 randomised (796 recruited) to blind group; 2159 randomised (1027 recruited) to non-blind group. | |
| Type of Recruitment Personnel | | | | | |
| Donovan et al 2003 (8) | Randomised trial nested within a trial of treatments for localised prostate cancer. | Men with localised prostate cancer. | Randomised to see a nurse or urologic surgeon for an 'information appointment' in which they were asked to consent to the ProtecT treatment trial. | Proportion in each group agreeing to treatment randomisation.  167 identified, 150 randomised to nurse or urologist (75 each). 50 randomised to trial treatment by nurse; 53 randomised to trial treatment by urologist | |
| Quinaux et al 2003 (9) | Methodological study performed in parallel with a phase IV trial comparing two chemotherapies. | 135 French trial centres. | Centres randomised to receive visits from monitors (reference arm), or not to receive visits (experimental arm). | Inclusion rate.  176 patients recruited in monitored centres versus 186 patients recruited in non-monitored centres. | |
|  | |  |  |  | |
| Table 1: Characteristics of included studies (cont). | |  |  |  | |
|  | |  |  |  | |
| ***Socio-cultural*** | | | | | |
| Ford et al 2004 (11) | Randomised trial with recruitment intervention designed to address 4 types of barriers to clinical trial participation. | African American men aged 55-74. | Randomised to one of three intervention arms (A, B or C) or the control arm (D).  A= enhanced recruitment letter by mail, telephone interview conducted by African American interviewer, baseline data collected by mail, reminders by telephone & mail.  B= enhanced recruitment letter by mail, telephone interview conducted by African American interviewer, baseline data collected by telephone, reminders by telephone & mail.  C= enhanced recruitment letter by mail, telephone interview conducted by African American interviewer, baseline data collected at church project session.  D= standard recruitment letter by mail, telephone interview conducted by African American or Caucasian interviewer, baseline data collected by mail, telephone & mail reminders | | Proportion of contacted and eligible men enrolling in the PLCO trial from each group  Of 39432 African American men, 17770 were able to be contacted and 12400 were eligible. Randomised to arms:  A (3079 with 78 eventually recruited),  B (3075 with 87 recruited),  C (2949 with 116 recruited) and  D (3297 with 95 recruited). |
| Larkey et al 2002 (10) | Randomised trial of recruitment within a longitudinal Women's Health Initiative (WHI) study. Recruitment trial was designed to test the effectiveness of selecting and training Hispanic WHI study participants to become lay advocates (Embajadoras) for the program. | Hispanic women and non-Hispanic white women  56 Hispanic WHI participants randomised to receive training (28) or no training (28 initially - 2 dropped out)  42 non-Hispanic white (Anglo) women assigned to control | Embajadoras = Hispanic WHI participants trained on advocacy, recruitment, trial design and concept and supplied with brochures and expression of interest cards  Hispanic controls= Hispanic WHI participants not trained, but supplied with brochures and expression of interest cards  Anglo controls = Non-Hispanic WHI participants, not trained, but supplied with brochures and expression of interest cards. | | Referral and enrolment activity  - Trained Hispanic women referred 31 women of whom 13 were recruited.  - Untrained Hispanic women referred 3 women of whom 0 were recruited.  - Untrained non-Hispanic white women referred 19 women of whom 2 were recruited. |
| Table 1: Characteristics of included studies (cont). | | | | | |
|  | | | | | |
| Strategies for contacting patients | | | | | |
| Aaronson et al 1996 (16) | Randomised study testing the efficacy of a supplementary, telephone based nursing intervention in increasing the patient’s awareness and understanding of the clinical trials in which they are asked to participate. | Cancer patients. 218 patients referred.  180 agreed and were randomised to two groups - 90 patients randomised to intervention group and 90 to the control group. | Control- standard informed consent procedures used in hospital phase II and III clinical trials.  Intervention- standard informed consent procedures supplemented by an informational contact with one or two oncology nurses | Evaluation of patients’ level of awareness  Patients’ anxiety level  Control group- 78 out of 90 eventually recruited.  Intervention group – 68 out of 90 eventually recruited | |
| Kendrick et al 2001 (14) | Randomised primary care prevention trial - response and recruitment rates to the trial using mass mailing comparing an invitation to participate with and without a home safety questionnaire. | Families with children under 5 years. | - Invitation to participate with home safety questionnaire, consent form, study information leaflet & freepost envelope  - Invitation to participate , consent form, study information leaflet & freepost envelope (i.e. no questionnaire) | Response and recruitment rates to the first mailing measured 3 weeks after sending the invitations.  1203 invited with questionnaire (259 responded with 217 recruited), 1190 invited without questionnaire (166 responded with 157 recruited) | |
| Kiernan et al 2000 (12) | Randomised trial examining dietary intervention. Recruitment study embedded within. | Hispanic men and women sent direct mail regarding intervention. | Randomly assigned to receive: Flyer about worksite dietary intervention or;  Same flyer plus a personalized hand-signed letter containing heart disease risk statistics for general American population or; Same flyer plus a personalized hand-signed letter containing heart disease risk statistics for Hispanics. | Response rate - percentage of individuals targeted by a recruitment strategy who responded and were eligible at initial contact.  Efficiency - percentage of individuals who responded and were eligible at the initial contact who were eventually randomised into the trial.  561 contacted:  191 with flyer alone, 187 ineligible or did not respond, 4 eligible.  370 with personalized letters, 341 ineligible or did not respond, 29 eligible (12 from group with general population risk statistics and 17 from group with Hispanic population risk statistics).  Of the 33 eligible respondents, 9 were randomised, all who had been sent on of the personalised letters. | |
|  |  |  |  |  | |
| Table 1: Characteristics of included studies (cont). | |  |  |  | |
|  |  |  |  |  | |
| *Strategies for contacting patients continued…* | |  |  |  | |
| Nystuen & Hagen 2004 (15) | Randomised controlled trial of a structured telephone follow-up versus no telephone follow-up to a recruitment letter into a community based trial. Intention to recruit analysis. Intention to phone analysis. | Sick listed employees | All eligible persons received a written invitation to participate in a study comparing standard treatment with a solution focused follow-up.  Those who did not respond within 2 weeks received either 'no reminder' (control) or 'attempted telephone reminder' (intervention). | Number of persons recruited to participate in the RCT.  703 randomised:  356 to intervention, 256 eventually received intervention from which 31 were recruited.  347 to control, 242 eventually received control from which 11 were recruited. | |
| Valanis et al 1998 (13) | Randomised chemoprevention trial for heavy smokers | Holders of health insurance plans who were to be recruited into Carotene and Retinol Efficacy Trial (CARET). | Sending of advance postcards one week prior to mailing the full recruitment pack;  Sending on the recruitment pack with no advance postcard | Proportion of respondents randomised.  11273 sent introduction postcard (225 randomised); 11273 not sent postcard (225 randomised) | |
| ***Trial administration*** | | | | | |
| Litchfield et al 2005 (17) | Trial comparing the efficiency and ease of the use of internet data capture compared with conventional paper based data recording in a clinical trial. Data management study embedded within a multi-centre, unblinded cluster randomised clinical trial of the effectiveness of two delivery systems for insulin. | Diabetic patients within 30 participating general practices. | Study centres were randomly allocated to use either the internet for data collection (internet system) or paper case report forms (paper system) | Timing for study landmarks, queries, data entry and time for monitoring  28 practices screened patients – 14 internet practices screened 52 patients (45 recruited) and 14 paper practices screened 28 patients (28 recruited) | |
| Incentives | | | | | |
| Martinson et al 2000 (18) | Randomised trial to evaluate the effect of monetary incentives on response rates of adolescents to a smoking-related survey as the first step to participation in an intervention trial. | 4200 adolescent members of a managed care organization randomised to one of 4 incentive groups. | Four incentive groups:  A $2 cash up front group;  A $15 cash on completion and return of survey group;  A $200 prize draw on completion and return of survey group;  A no-incentive control group. | Group specific response rate;  Subject consent to further contact.  4046 eligible:  996 in control group - 483 responded with 288 consenting to further contact.  3050 in other groups - 1960 responded with 1272 consenting to further contact. | |
